# Triple malignancy (NET, GIST and pheochromocytoma) as a first manifestation of neurofibromatosis type-1 in an adult patient

**DOI:** 10.1186/s13000-019-0848-7

**Published:** 2019-07-13

**Authors:** Karolina Poredska, Lumir Kunovsky, Vladimir Prochazka, Jiri Dolina, Miroslava Chovancova, Jakub Vlazny, Tomas Andrasina, Michal Eid, Petr Jabandziev, Petr Kysela, Zdenek Kala

**Affiliations:** 1 Department of Gastroenterology and Internal Medicine, University Hospital Brno, Faculty of Medicine, Masaryk University, Brno, Czech Republic; 2 Department of Surgery, University Hospital Brno, Faculty of Medicine, Masaryk University, Brno, Czech Republic; 3 Department of Pathology, University Hospital Brno, Faculty of Medicine, Masaryk University, Brno, Czech Republic; 4 Department of Radiology and Nuclear Medicine, University Hospital Brno, Faculty of Medicine, Masaryk University, Brno, Czech Republic; 5 Department of Hematology, Oncology and Internal Medicine, University Hospital Brno, Faculty of Medicine, Masaryk University, Brno, Czech Republic; 6 Department of Pediatrics, University Hospital Brno, Faculty of Medicine, Masaryk University, Brno, Czech Republic

**Keywords:** Neurofibromatosis type-1, von Recklinghausen disease, Neuroendocrine tumor, Gastrointestinal stromal tumor, Pheochromocytoma

## Abstract

**Background:**

Neurofibromatosis type-1 (NF1), also called von Recklinghausen disease, is a rare genetic disease which can lead to the development of benign or even malignant tumors. NF1 is mostly diagnosed in children or early adolescents who present with clinical symptoms. A curative therapy is still missing and the management of NF1 is based on careful surveillance. Concerning tumors which affect the gastrointestinal tract in patients with NF1, the most common is a gastrointestinal stromal tumor (GIST).

**Case presentation:**

We present a case of a 58-year-old adult patient with dyspeptic symptoms who was incidentally diagnosed with triple malignancy (pheochromocytoma, multiple GISTs of small intestine and an ampullary NET) as a first manifestation of NF1. The patient underwent surgical treatment (adrenalectomy and pancreaticoduodenectomy) with no complications and after 2 years remains in oncological remission.

**Conclusion:**

NF1 is a rare genetic disease which can cause various benign or malignant tumors. The coincidence of GIST and NET is almost pathognomonic for NF1 and should raise a suspicion of this rare disorder in clinical practice.

## Introduction

Neurofibromatosis type-1 (NF1), also called von Recklinghausen disease, is a rare autosomal dominant disorder which can be either inherited or sporadic. The incidence of NF1 is approximately 1 in 3000 individuals [1]. NF1 is caused by a mutation in the tumor suppressor gene NF1 which leads to a decreased production of the protein neurofibromin and subsequently to the risk of developing benign or malignant tumors with a variety of clinical symptoms [2]. The definitive diagnosis is made if the patient meets a minimum of two of the following criteria: 6 or more cafe-au-lait macules larger than 5 mm before puberty or 15 mm after puberty, skinfold freckling, 2 or more neurofibromas or 1 plexiform neurofibroma, 2 or more Lisch nodules, optic glioma, characteristic skeletal dysplasia and affected first-degree relative [3, 4]. NF1 is most frequently diagnosed in children or adolescents [3]. When diagnosed in adulthood the clinical symptoms mainly include neurofibromas, cognitive deficit, pheochromocytoma, malignant peripheral nerve sheath tumor (MPNST), vascular dysplasia and hypertension [2].

Patients with NF1 are at an increased risk of malignancy, especially MPNST, leukemia and rhabdomyosarcoma [3]. The frequency of tumors affecting the gastrointestinal tract in NF1 patients is difficult to assess, however the most common is gastrointestinal stromal tumor (GIST) with an incidence which varies from 5 to 25% [5, 6]. According to the literature, the coincidence of GIST and periampullary neuroendocrine tumor (NET) is almost pathognomonic for NF1 diagnosis [7]. Concerning the management of patients with NF1, there is unfortunately no curative therapy and the patients have to undergo regular check-ups for any complications [2–4].

## Case report

**Fig. 1:**
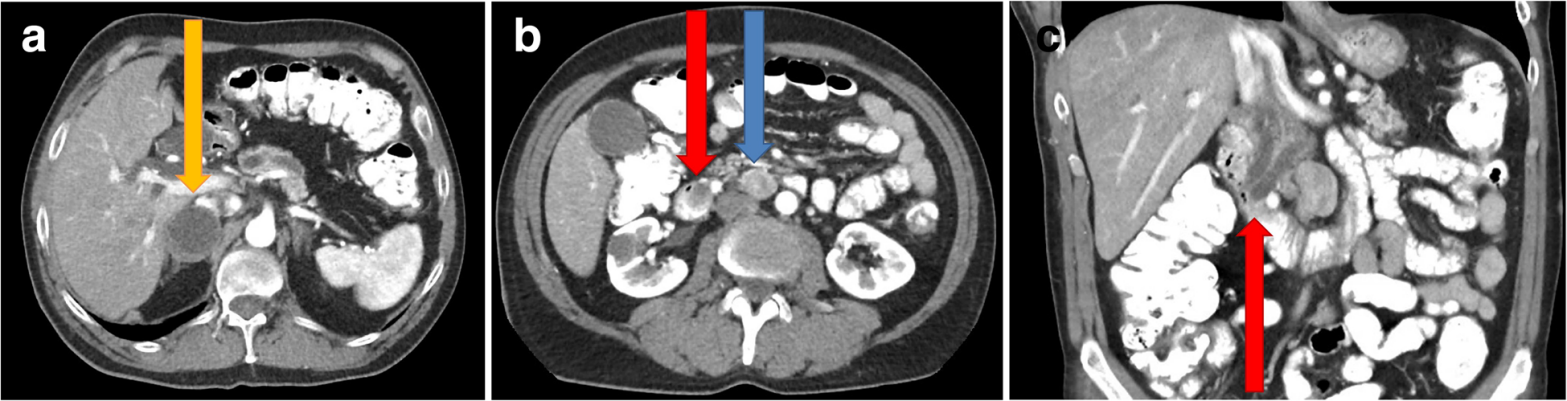
**a** Abdominal CT (transversal scan) - expansion of the right adrenal gland 4 × 4 cm in size (orange arrow). **b** Abdominal CT (transversal scan) - tumor of the ampulla of Vater (red arrow) with associated lymphadenopathy (blue arrow). **c** Abdominal CT (coronal scan) - tumor of the ampulla of Vater (red arrow) with a double-duct sign

We present a case report of a 58-year-old patient with a history of chronic pancreatitis, spontaneous pneumothorax due to bullous emphysema, combined pulmonary fibrosis, hypertension, and congenital left eye blindness. He denied any allergies and regularly used pancreatic enzymes and antihypertensive medication. Concerning his family history, his father died of an unspecified renal disease. Due to repetitive dyspepsia the patient underwent a basic examination (laboratory findings, abdominal ultrasound) with no diagnostic findings. The abdominal computed tomography (CT) scan revealed an infiltration of the right adrenal gland (4 × 4 cm in size) and an infiltration of the ampulla of Vater (with a double-duct sign) with associated lymphadenopathy (Fig. 1a, b, c). A gastroscopy showed an enlarged, solid ampulla of Vater, histologically described as a duodenal NET, grade 1. The endoscopic ultrasound confirmed the infiltration of the ampulla of Vater extending into the duodenal wall (2 cm in size).

**Fig. 2:**
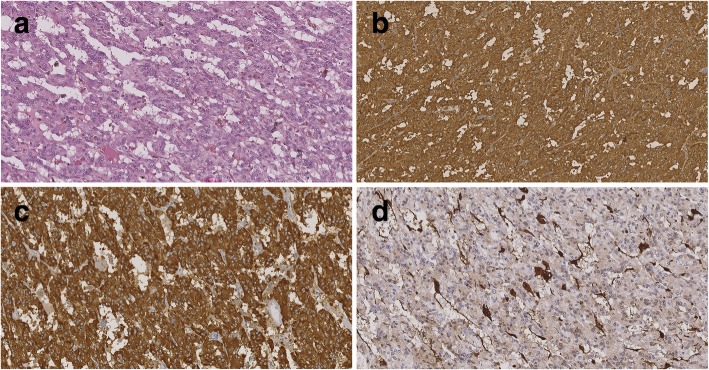
**a** Histopathology - pheochromocytoma, tumor with a trabecular pattern of polygonal cells set in rich vascular network. Cells have finely granular cytoplasm, oval nuclei with prominent nucleoli and a coarsely clumped chromatin, without mitotic figures, HE staining, 200x. **b** Histopathology - pheochromocytoma, diffuse strong positivity of tumor cells, chromogranin, 200x. **c** Histopathology - pheochromocytoma, diffuse strong positivity of tumor cells, synaptophysin, 200x. **d** Histopathology - pheochromocytoma, positivity of the sustentacular cells at the periphery of the tumor cell clusters, S100 protein, 200x

**Fig. 3:**
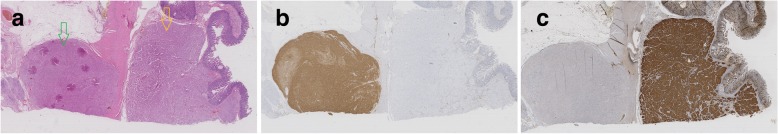
**a** Histopathology - GIST (green arrow) and NET (yellow arrow), HE staining, 10x. **b** Histopathology - diffuse positivity of GIST, negativity of NET, CD117 (c-kit), 10x. **c** Histopathology - GIST negative, NET diffusely positive, chromogranin, 10x

**Fig. 4:**
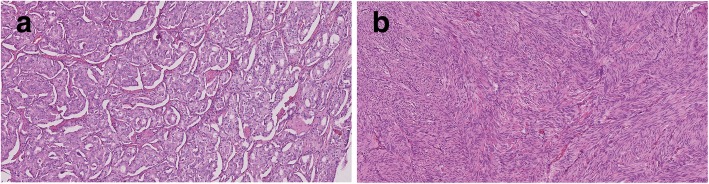
**a** Histopathology - NET G1, tumor with solid and nesting masses of monotonous small round cells. Cells have moderate amount of finely granular cytoplasm, small nucleoli and “salt and pepper” chromatin pattern. No mitotic figures are seen, HE staining, 100x. **b** Histopathology - GIST, the tumor cells show plump spindled cell morphology with minimal atypia, without mitotic figures. The cells are arranged in whorls or short intersecting fascicles, with frequent and prominent nuclear palisading, HE staining, 100x

**Fig. 5:**
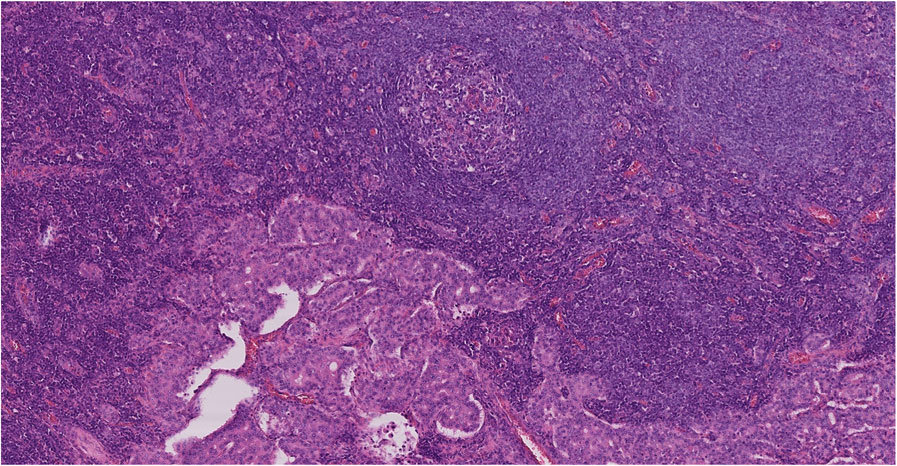
Histopathology - lymph node with a metastasis of NET, HE staining, 100x

According to the multidisciplinary (gastroenterologist, oncologist, radiologist and surgeon) committee’s decision, the patient was advised to undergo a surgical treatment (adrenalectomy and pancreaticoduodenectomy). The histopathological results showed pheochromocytoma of the adrenal gland (Fig. 2a, b, c, d), NET of the ampulla of Vater (grade 1 with invasion to the duodenum, T2 N1, lymph nodes 7/14) and, surprisingly, multiple GISTs (T1 N0) located in the proximal duodenum (5 mm in size), in the duodenum (7 mm in size) and in the proximal jejunum (7 mm in size) (Figs. 3a, b, c, 4a, b and 5). There were no post-surgical complications and the patient was released 10 days after the procedure. We completed a genetic examination which revealed a mutation in the NF1 gene (c.1570G-Tp.Glu524*). After an additional, thorough clinical examination, typical café-au-lait macules and small neurofibromas on the chest, back and arms were also found. Two years later, the patient is in oncological remission with regular surveillance by an oncologist, dermatologist, ophthalmologist and neurologist.

## Discussion

According to the guidelines for the diagnosis and management of NF1, the typical age of onset of the major clinical manifestations is in childhood or adolescence; however, our patient was 58 years old at the time of diagnosis [3, 4]. Moreover, according to the guidelines the clinical assessment should be sufficient to confirm the diagnosis. Upon clinical examination, our patient showed only café-au-lait macules, but these patches can also occur in 10% of the general population [3]. There is a possibility of a positive family history in our case, but this cannot be confirmed by the patient. Therefore, our patient did not meet the clinical diagnostic criteria.

However, the first manifestation of NF1 in our patient was an incidental diagnosis of a triple malignancy (pheochromocytoma, ampullary NET and GIST of duodenum and jejunum), which corresponds with the fact that the gastrointestinal manifestations of NF1 are often under-diagnosed [7]. The GISTs associated with NF1 are typically multiple, located in the stomach, duodenum or proximal jejunum, and are mainly diagnosed incidentally [6]. The presence of multiple GISTs was also reported in our patient. The treatment of GISTs is preferably surgical, either by pancreaticoduodenectomy or a segmental resection of the duodenojejunal portion [8, 9]. The finding of GIST and NET in one patient is supposed to be almost diagnostic for NF1 [7]. NETs of the ampullary region are mainly solved by pancreaticoduodenectomy. In cases of NETs smaller than 2 cm with no lymph node involvement an endoscopic resection or transduodenal surgical ampulectomy might be the method of choice [5, 10, 11]*.* In our case a pancreaticoduodenectomy was performed to resect both the GISTs of the duodenum and jejunum and the ampullary NET. Concerning the pheochromocytoma, its most common clinical manifestation are episodes characterized by palpitations, diaphoresis, headaches and severe hypertension [12]. However, our patient had hypertension which reacted well to basic antihypertensive therapy (amlodipine) with no episodes. In the treatment of pheochromocytoma, the method of choice is a surgical resection, which was also performed in the case of our patient [12].

As far as the surveillance of patients with NF1 is concerned, they should be examined annually by a physician and sent to a specialist if complications such as rapidly growing or painful skin lesions, headaches, nerve pain and visual disturbances occur [13].

## Conclusion

NF1 is a rare genetic disorder diagnosed mainly in children. Here we present a case report of a male adult patient with triple malignancy (pheochromocytoma, GISTs of the small intestine and ampullary NET) as a first incidental manifestation of NF1. As our case report indicates, diagnosing GIST and NET in one patient is almost pathognomonic for NF1.

## Data Availability

As a case report, all data generated or analyzed are included in this article.

## Abbreviations

CT: Computed tomography
GIST: Gastrointestinal stromal tumor
MPNST: Malignant peripheral nerve sheath tumor
NET: Neuroendocrine tumor
NF1: Neurofibromatosis type-1
